# Assessment of sleep quality and hypothyroidism among medical students in India

**DOI:** 10.6026/973206300221018

**Published:** 2026-02-28

**Authors:** Avijit Mondal, Oona Chakrabarty, Mandrita Chatterjee

**Affiliations:** 1 Department of Physiology, Dr. B.C. Roy Multi-Speciality Medical Research Centre, IIT Kharagpur, India; 2 Department of Physiology, Diamond Harbour Government Medical College and Hospital, Diamond Harbour, India

**Keywords:** Thyroid-Stimulating Hormone (TSH), FT4, sleep quality, Pittsburgh sleep quality index (PSQI), Subclinical hypothyroidism

## Abstract

Hypothyroidism reduces thyroid hormone levels, slowing metabolism and is prevalent in India. Therefore, it is of interest to relate hypothyroidism and poor sleep among first-year medical students in eastern India. Hence, an Observational Cross-sectional study was conducted on apparently healthy students aged 18-25 years of a Medical College of Eastern India. Socio demographics, anthropometric measurements, Free T4, TSH and Sleep quality (PSQI) were assessed. 17.241% students had Hypothyroidism, 9.195% students had Subclinical Hypothyroidism, 44.83% students were found to have poor sleep. Female students had significantly more FT4, TSH and poorer sleep quality than male students. (p=0.0282, p=0.0111 and p=0.0094). A strong positive correlation was found between serum TSH levels and PSQI scores (R =0.786). Thus, there is a significant burden of hypothyroidism, subclinical hypothyroidism and poor sleep quality among medical students. There is also a strong association between elevated serum TSH levels and impaired sleep quality.

## Background:

Hypothyroidism, a disease caused by decreased metabolism or decreased synthesis and secretion of thyroid hormones is common in India. It affects approximately 1 in 10 adults (approximately 11%), with prevalence significantly higher among women and the elderly and is often associated with iodine deficiency and autoimmune factors [1]. Studies show that the overall prevalence is approximately 10.95%, with a significant proportion being undiagnosed. Subclinical hypothyroidism is also common (8.02%) [2]. Poor sleep quality has negative effects on endocrine, immune, cardiovascular, neurological and cognitive functions and may also lead to the onset and development of chronic diseases [3- 4]. Current research shows that hypothyroidism can cause sleep disorders such as obstructive sleep apnea, restless legs syndrome and daytime sleepiness. Sleep dysfunction is also responsible for disorders of thyroid function [5, 6-7]. For example, Zhou *et al.* explored the links between thyroid function, metabolic health, and sleep apnea (OSA) [8]. Low thyroid hormone levels can disrupt sleep-wake cycle, leading to difficulty falling asleep, more frequent night awakenings and shorter sleep duration [9]. Medical students and other health care professionals receive insufficient attention in the early diagnosis and prevention of many diseases. Thus, early identification of medical students suffering from Hypothyroidism is crucial. Therefore, it is of interest to assess sleep and hypothyroidism in first-year medical students in eastern India and to clarify their relationship.

## Methodology:

Study commenced after obtaining the ethical clearance from Institutional Ethics Committee of the concerned Government Medical College & Hospital, West Bengal;

[1] Study Type: Descriptive, Epidemiological, institution-based

[2] Study Design: Cross-Sectional

[3] Sampling Design: Complete Enumeration

[4] Study population: Apparently healthy Male and Female Students without any symptoms aged 18-25 years of a Government Medical College & Hospital in Easten India

## Inclusion criteria:

Apparently healthy Male and Female Students aged 18-25 years who volunteered to participate in the study.

## Exclusion criteria:

[1] Participants suffering from cardiovascular disease or congenital heart disease

[2] students who were suffering from chronic diseases,

[3] Persons with any history of Hypo or Hyperthyroidism

[4] Chronic alcoholism and malignancy.

## Study area:

Department of Physiology & Biochemistry of a Government Medical College & Hospital, West Bengal, India

## Study duration:

6 months

## Recruitment of the participants:

All healthy young medical students between the age group of 18 to 25 years were included in the study based on inclusion and exclusion criteria

## Tools/ Description of procedure:

The study was conducted as per the WHO STEPwise approach for surveillance of risk to non-communicable diseases. The three levels of the questionnaire, physical measurements and biochemical measurements were adhered to during investigation [10]. A semi-structured questionnaire for socio-demographic details of subjects like age, gender, physical activity, family history was procured. All the students were subjected to thyroid assay like FT4, TSH levels. After taking general history, clinical examination, anthropometric (height, weight, BMI, Waist circumference, hip circumference) and Blood pressure were assessed. Blood was collected by trained phlebotomist at early morning and serum (100-200 μl) was used for the hormone assay immediately after centrifugation. Hormone assay \ for TSH was done by Chemiluminescence immunoassay (CLIA). Participants with serum free T4 <0.89 ng/dl and TSH >5.50 μU/ml, were categorized as hypothyroid. Elevated TSH with normal T4 was classified as subclinical hypothyroidism [11]. Pittsburgh Sleep Quality Index (PSQI) was selected as the data collecting tool for assessing sleep quality. The PSQI is a subjective measurement tool that analyses the quality of sleep within one month. The Indian version of the PSQI was applied. Each of the seven components of the PSQI is awarded a score ranging between 0 and 3 points, with the sum of these values constituting a global score, varying from 0 to 21 points. A global score of >6 was the optimal cut-off point for distinguishing Normal Sleepers (NS) and Poor Sleepers (PS) students [12]. For diagnosing obesity, the waist circumference cutoff for Asians was used. Accordingly, there is a cutoff value of ≥90 cm for males and ≥80 cm for females for central obesity [13]. A body mass index (BMI) of ≥25 kg/m^2^ served as an indicator for diagnosing obesity, for both males and females [14]. Data analysis was carried out using SPSS version 25 software. Appropriate Statistical tests were used to analyse the data available. Results were considered significant if p<0.05.

## Results and Discussion:

**Figure 1:**
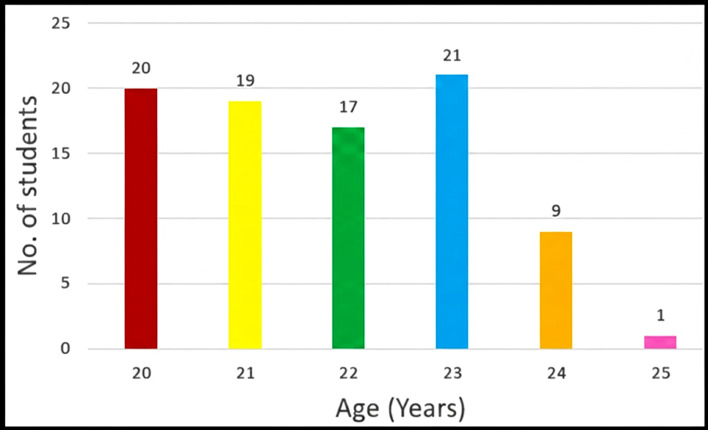
Bar diagram showing distribution of participants according to age

The total number of students participating in the study was 87. Most of the students did not have any family history of hypothyroidism (Table 1). The Mean age of the participants was 21.805± 1.371 years; Most of the students were 20 years of age (Figure 1). 33 students (37.93%) were obese according to the Asian cut off for BMI (≥25 kg/m^2^). According to Waist circumference, 17 females (48.57%) belonged to obese category, whereas 10 males (19.23%) were found to be obese (Table 2).

**Figure 2:**
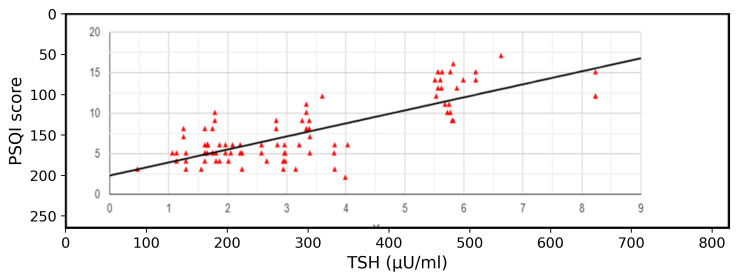
Graph showing correlation of TSH level and Sleep Quality (PSQI score) according to Pearson's Correlation

The students spent 6.87 ± 0.69 hours in bed, actually sleeping for an average of 6.47 ± 0.65 hours. Pittsburgh Sleep Quality Index (PSQI) was selected as the data collecting tool for assessing sleep quality. 39 (44.83%) students were found to have poor sleep (Table 3). Participants with serum free T4 <0.89 ng/dl and TSH >5.50 μU/ml, were categorized as hypothyroid. Elevated TSH with normal T4 was classified as subclinical hypothyroidism [11]. 15 (17.241%) students had Hypothyroidism based on free T4 and TSH level. 8 (9.195%) students had Subclinical Hypothyroidism (Table 4). When comparing male and female students, there were no significant differences in age (p=0.7472), waist circumference (p=0.8876), hip circumference (p=0.8255), weight (p=0.107). However, Males had significantly more height than the females (p<0.0001). Both Systolic and Diastolic Blood pressure were significantly higher in males than females (p=0.0469 and p<0.0001). But Females had significantly more FT4, TSH and PSQI score (Poor sleep) than the males (p=0.0282, p=0.0111 and p=0.0094) (Table 5). In case of Physical Activity, most males (18) were in Mild Exercise category. On the other hand, Equal no of females (13) were in No exercise and Moderate Exercise category. 8 Males (15.69%) and 13 females (36.11%) had history of Hypothyroidism in their families. 4 males (7.84%) and 11 females (30.56%) had Hypothyroidism, whereas 5 males (9.8%) and 3 females (8.33%) were found to have subclinical hypothyroidism. Among Males, 18 (35.29%) were found to be Poor sleepers according to PSQI score, whereas 21 females (58.33%) were Poor sleepers. Compared to males, there were no significant differences in Physical activity (p=0.44) in case of females. However, significantly more Female students had family history of Hypothyroidism (p= 0.0283), were found to have Hypothyroidism and Subclinical Hypothyroidism (p=0.0217) and were suffering from poor sleep (p=0.0333), compared to the male students (Table 6). Pearson's correlation test was used to find the association between serum TSH level and sleep quality (PSQI score) of the medical students. The value of R is 0.786. This is a strong positive correlation, which means that high serum TSH levels go with High PSQI scores (and vice versa). The p value is <0.00001 (Figure 2).

This cross-sectional study reveals a significant prevalence of thyroid dysfunction among medical students, accompanied by poor sleep quality. Hypothyroidism was identified in 17.24 % of participants, with subclinical hypothyroidism accounting for 9.19%. These suggest that thyroid dysfunction is common in young adults exposed to sustained academic stress and lifestyle disruption. The prevalence observed in the present study is substantially higher than that reported by Kadel *et al.* among female medical students in Nepal, where a prevalence of only 2.12 % was documented [15]. The lower prevalence in that study may be attributed to differences in study methodology and reliance on symptom-based screening followed by selective biochemical testing. In contrast, the present study employed biochemical evaluation of thyroid function in all participants, which likely improved the detection of both overt and subclinical hypothyroidism. This comparison highlights the limitation of symptom-based screening in young populations. Both studies demonstrate that hypothyroidism is not uncommon in young women. The higher prevalence observed among medical students may reflect additional risk factors such as academic stress, irregular sleep schedules and lifestyle imbalance. This study also highlighted that positive family history of thyroid illness is a major risk factor for developing hypothyroidism. In Studies among college students show that those with an affected relative have a significantly increased risk of developing the condition themselves. Siblings each had a 6-fold higher risk, while children had a 3-fold higher risk of developing hypothyroidism [16]. In our study, we found 21 (24.18%) students with positive history of hypothyroidism in the family. Subclinical hypothyroidism formed a considerable proportion of thyroid dysfunction in the present study. It is a finding that closely parallels the observations of a previous study, who reported an 18 % prevalence of subclinical hypothyroidism in Gujrat, associated with metabolic syndrome [17]. Previous studies emphasise that subclinical hypothyroidism is common even in young, healthy college students [18]. One of the most impactful findings of the present study is the strong positive correlation between serum TSH levels and PSQI scores, indicating that poorer sleep quality is associated with higher TSH concentrations. This observation is strongly supported by the NHANES-based study by Ding *et al.* It demonstrated that sleep disturbances and poor sleep patterns were independently associated with increased odds of hypothyroidism after adjustment for demographic, metabolic and autoimmune factors [19]. While the NHANES study examined a broad adult population, the present findings confirm that this association is evident even in young adults, suggesting that sleep quality may influence thyroid function early in life. The mechanistic basis for this association is biologically plausible. Thyroid-stimulating hormone secretion follows a circadian rhythm and disruption of normal sleep architecture may alter hypothalamic-pituitary-thyroid axis regulation. Poor sleep quality is also known to affect immune and inflammatory pathways, potentially contributing to thyroid dysfunction, particularly in individuals with genetic susceptibility. Although hypothyroidism itself can impair sleep, the strong correlation observed in this largely asymptomatic population suggests that sleep disturbance may act as a contributing factor rather than solely a consequence of overt disease. The gender-based differences observed in the present study further reinforce the existing literature. Female students exhibited significantly higher TSH levels, poorer sleep quality and a higher prevalence of family history of hypothyroidism. The clustering of poor sleep quality and thyroid dysfunction among female students in the present study underscores the importance of early, gender-sensitive screening strategies in high-stress academic environments.

## Strength and weakness:

The strengths of this study include complete biochemical assessment of thyroid function in all participants and use of a validated tool for assessing sleep quality. However, the cross-sectional design limits causal inference and the findings from a single institution may restrict generalizability. Additionally, sleep quality was assessed subjectively and objective sleep measurements were not employed.

## Conclusion:

There is a significant burden of hypothyroidism and subclinical hypothyroidism among medical students along with a high prevalence of poor sleep quality. It suggests that thyroid dysfunction may remain clinically silent without biochemical screening is reported. The predominance of subclinical disease and the higher vulnerability observed among female students emphasize the need for early, targeted screening in academically stressed populations. Integrating periodical biochemical thyroid screening with routine sleep assessment may support early identification and preventive strategies.

## Advancement to knowledge:

This study explores the relationship between sleep quality and hypothyroidism among Indian medical students, an under-researched area. By generating India-specific data on sleep patterns and thyroid status, it may identify undiagnosed thyroid dysfunction contributing to fatigue and poor performance. The findings can support early screening, improve student wellness programs and enhance understanding of sleep-endocrine interactions in young adults.

## Figures and Tables

**Table 1:** Distribution of participants according to their gender, physical activity and family history

**Characteristics**	**Frequency (N=87)**
**Gender**	
Male	52
Female	35
Physical activity	
No Exercise (Sedentary)	27
Mild Exercise	26
Moderate Exercise	27
Vigorous Exercise	7
**Family History of Hypothyroidism**	
No Hypothyroid in family	66
Family history of Hypothyroid present	21

**Table 2:** Anthropometrics and blood pressure data of the participants

**Characteristics**	**Mean**	**SD**
Height (cm)	165.954	7.635
Weight (kg)	65.741	10.694
Waist Circumference (cm)	80.276	10.793
Hip Circumference (cm)	90.494	9.999
Systolic Blood Pressure (mm Hg)	120.184	9.625
Diastolic Blood Pressure (mm Hg)	79.667	7.227

**Table 3:** Sleep Patterns of medical students according to PSQI scale

**Sleep Pattern**	**Count**	**PSQI Score**
Poor Sleeper	39	7.678 ±3.856
Normal Sleeper	48	

**Table 4:** Biochemical assessment of the participants

**Parameters**	**Mean**	**SD**
FT4 (ng/dl)	1.274	0.299
TSH (μU/ml)	3.341	1.888

**Table 5:** Comparison of the risk factors according to genders

**Parameters**	**Group**	**Mean**	**N**	**Std. Deviation**	**P value**
Age	Male	21.765	51	1.45	0.7472
	Female	21.861	36	1.268	
Waist Circumference	Male	80.137	51	9.881	0.8876
	Female	80.472	36	12.11	
Hip Circumference	Male	90.294	51	9.455	0.8255
	Female	90.778	36	10.855	
Height	Male	169.843	51	5.057	<0.0001
	Female	160.444	36	7.311	
Weight	Male	67.294	51	9.244	0.107
	Female	63.542	36	12.371	
SBP	Male	121.902	51	10.887	0.0469
	Female	117.75	36	6.921	
DBP	Male	82.118	51	6.062	<0.0001
	Female	76.194	36	7.394	
FT4	Male	1.333	52	0.272	0.0282
	Female	1.191	35	0.319	
TSH	Male	2.913	52	1.852	0.0111
	Female	3.946	35	1.79	
PSQI Score	Male	6.784	52	3.331	0.0094
	Female	8.944	35	4.229	
*p<0.05 taken as significant

**Table 6:** Comparison of physical activity, family history, hypothyroidism and sleep quality among males and females

**Parameters**			**Group**		**Total**
			Male	Female	
Physical Activity	No Exercise	Count	14	13	27
		% within Group	51.85%	48.15%	100%
	Mild Exercise	Count	18	48.15%	26
		% within Group	69.23%	30.77	100%
	Moderate Exercise	Count	14	13	27
		% within Group	51.85%	48.15%	100%
	Vigorous Exercise	Count	5	2	7
		% within Group	71.43%	28.57%	100%
P value		0.44			
Chi square		2.7			
Family History of Hypothyroidism	No Hypothyroid in Family	Count	43	23	66
		% within Group	65.15%	34.85%	100%
	Family history of Hypothyroid present	Count	8	13	21
		% within Group	38.10%	61.90%	100%
P value		0.0283			
Chi square		4.8078			
Hypothyroidism	Hypothyroid	Count	4	11	15
		% within Group	26.67%	73.33%	100%
	Subclinical Hypothyroid	Count	5	3	8
		% within Group	62.50%	37.50%	100%
	Euthyroid	Count	42	22	64
		% within Group	65.63%	34.37%	100%
P value		0.0217			
Chi square		7.6581			
Sleep Quality	Poor Sleeper	Count	18	21	39
		% within Group	46.15%	53.85%	100%
	Normal Sleeper	Count	33	15	48
		% within Group	68.75%	31.25%	100%
P value		0.0333			
Chi square		4.5292			
*p<0.05 taken as significant
